# Metabolomic Fingerprinting and Molecular Characterization of the Rock Samphire Germplasm Collection from the Balkan Botanic Garden of Kroussia, Northern Greece

**DOI:** 10.3390/plants11040573

**Published:** 2022-02-21

**Authors:** Kalliopi Kadoglidou, Maria Irakli, Anastasia Boutsika, Ifigeneia Mellidou, Nikolas Maninis, Eirini Sarrou, Vasiliki Georgiadou, Nikolaos Tourvas, Nikos Krigas, Theodoros Moysiadis, Katerina Grigoriadou, Eleni Maloupa, Aliki Xanthopoulou, Ioannis Ganopoulos

**Affiliations:** 1 Institute of Plant Breeding and Genetic Resources, ELGO-DIMITRA, Thermi, GR-57001 Thessaloniki, Greece; irakli@cerealinstitute.gr (M.I.); bouanastasia@outlook.gr (A.B.); ifimellidou@gmail.com (I.M.); nikolasmaninis@gmail.com (N.M.); esarroy@gmail.com (E.S.); vaso.geo@gmail.com (V.G.); nikostourvas@gmail.com (N.T.); nikoskrigas@gmail.com (N.K.); moysiadis.t@unic.ac.cy (T.M.); kgrigoriadou@ipgrb.gr (K.G.); maloupa@bbgk.gr (E.M.); aliki.xanthopoulou@gmail.com (A.X.); 2 Department of Computer Science, School of Sciences and Engineering, University of Nicosia, Nicosia 2417, Cyprus

**Keywords:** *Crithmum maritimum*, phenolics, ascorbic acid, antioxidant capacity, genetic diversity, molecular markers

## Abstract

The traditionally edible aerial parts of rock samphire (*Crithmum maritimum* L.) could be a valuable functional food or feed ingredient due to their high antioxidant capacity, ascorbic acid content, and rich content in secondary metabolites such as phenolics and flavonoids. The first objective of this study was to evaluate eighteen genotypes derived from different regions of Greece regarding the phytochemical contents of their soluble extracts in total phenolics, total flavonoids, and individual polyphenols as determined by LC-MS analysis, as well as ascorbic acid content and their antioxidant capacity as determined by different assays, including ABTS (2,2-azino-bis-3-ethylbenzothiazoline-6-sulfonic acid), DPPH (2,2-diphenyl-1-picrylhydrazyl radical scavenging activity), and FRAP (ferric reducing antioxidant power) assays. The second objective of the study was the molecular characterization of native Greek *C.* *maritimum* genotypes. Great variation among genotypes was observed in terms of the antioxidant capacity, ascorbic acid content, and phenolic compounds (total phenolic content and total flavonoid content), as well as in caffeolquinic acids and flavonoids. The principal component analysis highlighted genotypes with a higher potential in antioxidants and polyphenolics. The most promising genotypes were G9 from Kefalonia, followed by G4 from Ikaria, where both clearly exhibited a similar response with high values of evaluated traits. The molecular characterization of genotypes revealed low variability and low to moderate genetic diversity between populations. Our data indicated that the rock samphire germplasm collection from the Balkan Botanic Garden of Kroussia could serve as an important source of documented genetic material and, thus, it is suggested for further investigation to provide insight regarding cultivation and agro-processing aspects, artificial selection, or plant breeding aimed at developing *C.* *maritimum* genotypes of high-bioactive value.

## 1. Introduction

*Crithmum maritimum* L., also known as rock samphire or sea fennel, is a native Greek, wild-growing plant of the Apiaceae family. It is a perennial, medicinal-aromatic plant with fleshy leaves that are traditionally edible [1]. Rock samphire naturally thrives on rocky crevices as a chasmophyte (rock-dweller) and in sandy substrates as a halophyte [2]. *C. maritimum* has a Euri-Mediterranean native distribution, extending to the Atlantic European coastline and the British Isles as well as along parts of the Black Sea coastline [3,4]. At the beginning of the twentieth century, *C. maritimum* has also been established as an alien plant along the coasts of Belgium and the Netherlands, mainly in disturbed man-made environments such as harbors and dikes [5].

Rock samphire attracts considerable scientific interest, mainly due to its properties. Rock samphire is used as a food ingredient in several traditional recipes [6] and it is known for its good sensory traits due to its high essential oil content [7]. Moreover, as a food ingredient, it is associated with positive health effects [8] due to its richness in biologically active compounds in the leaves, such as vitamin C, iodine, carotenoids, flavonoids, minerals (mainly calcium), organic acids, and phenolics [9,10], whereas the seeds are beneficial for human consumption as they are rich in essential fatty acids [11]. The most commonly detected phenolic compounds of rock samphire are phenolic acids such as chlorogenic, neochlorogenic, cryptochlorogenic, ferulic, and caffeoylquinic acids and its derivatives [12]. This edible halophyte also contains interesting amounts of gallic, caffeic, vanillic, rosmarinic, and *p*-coumaric acids, while small amounts of trans-2-hydroxycinnamic and trans-cinnamic acids have also been reported [12]. Moreover, flavonoids such as rutin, apigenin, quercetin-3-galactoside, epicatechin, epigallocatechin, catechin, pyrocatechol, and 4-hydroxybenzaidehyde (polyphenols) are also present in *C. maritimum*, but in rather low amounts [13,14].

Innovation-wise, the encapsulation of rock samphire’s essential oil is reported as a promising alternative for the control of insects causing human diseases (e.g., the dengue vector *Aedes aegypti*) and crop pests (e.g., the cotton leafworm *Spodoptera litura*) [15]. Additionally, rock samphire may be an alternative for both horticultural and industrial crops in the case of low soil quality and/or irrigation water with high electrical conductivity [6], whereas, at the same time, methods for its production with compost-based substrates has recently been investigated [10].

A survey of the literature makes evident that the primary focus of most previous investigations mainly concern the phytochemical profile and the biological activities of *C. maritimum* e.g., [16,17,18], as well as the growing techniques of rock samphire e.g., [10,19,20]. Nevertheless, to the best of our knowledge, comparative studies are scarce regarding the bioactive compounds, the antioxidant capacity, the metabolomic analysis, and the genetic identification of different rock samphire genotypes/ecotypes, and this research field remains almost unexplored e.g., [5,21,22]. Generally, the identification of genetic variation could be based on the changes accrued on nucleotide sequences of DNA stands during the process of genome proliferation. Various techniques have been developed to identify the species diversity at the molecular level, such as genetic markers. These markers are necessary tools for the investigation of the intraspecific genetic diversity across the geographical distribution of plant species. In general, these markers mainly differ in their application requirements, sensitivity, and the degree of reliability [23]. A highly reproducible polymorphic DNA fingerprinting technique based on polymerase chain reaction (PCR) is the Amplified Fragment Length Polymorphism (AFLP) [24]. The AFLP has been applied for the discrimination of genetic diversity in various species [25,26]. The Inter-Simple Sequence Repeat (ISSR) is another molecular marker that involves PCR amplifications of DNA [27]. It has proven to be a rapid, simple, and inexpensive technique to analyze the structure and genetic diversity of species, as well as to analyze the genetic relationships among cultivars [28]. With respect to rock samphire, previous studies [5] have developed nuclear microsatellite loci by using 454 pyrosequencing, and they have shown a strictly coastal geographical distribution with high levels of genetic differentiation (FST = 0.3) and a genetic structure typical of a mostly selfing species [unbiased fixation index (FIS) ranging from 0.16 to 0.58].

Taking all of this information into account, the aim of this study was to provide insight through a comparative analysis of the genetic diversity, the polyphenolic profile, and the antioxidant capacity of native Greek rock samphire genotypes.

## 2. Results and Discussion

A total of 18 rock samphire accessions with geographic origin from 8 different and representative regions of Greece are presented with relative information in Table 1.

### 2.1. Antioxidant Activity, Ascorbic Acid Content, Total Phenolic Content, and Total Flavonoid Content

An analysis of variance (ANOVA) applied to data obtained from antioxidant capacity determined with three different assays [2′-azinobis-(3-ethylbenzothiazoline-6-sulfonate) radical scavenging activity (ABTS), 2,2-diphenylpicrylhydrazyl radical scavenging activity (DPPH), and ferric reducing antioxidant power assay (FRAP)], as well as on ascorbic acid (AsA) content, showed a significant effect due to tested genotype of rock samphire as source of variation (Table 2).

**Figure 1:**
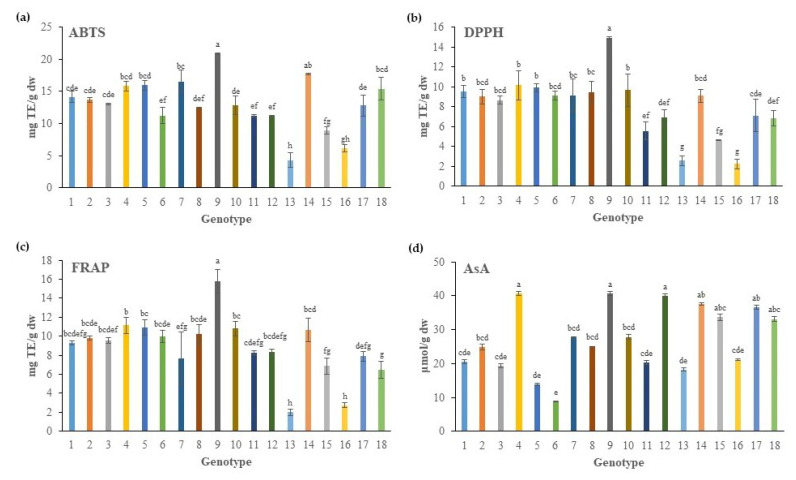
Overview of the antioxidant capacity of extracts derived from the aboveground portion of 18 native Greek rock samphire genotypes (Table 1) determined as 2,2-azino-bis-(3-ethylbenzothiazoline-6-sulfonic acid) radical scavenging activity (ABTS) (**a**); 2,2-diphenyl-1-picrylhydrazyl radical scavenging activity (DPPH) (**b**); and ferric reducing antioxidant power (FRAP) (**c**); all values are expressed as mg of trolox equivelant (TE) per g of dry weight (dw). The content of ascorbic acid (AsA), expressed as μmol of AsA per g of dw, is also shown (**d**). Data represent the mean values ± standard deviation. Different letters on the bars characterize significant differences among genotypes according to Tukey’s test for *p* ≤ 0.05.

Comparing the various genotypes, the G9 genotype clearly presented the highest antioxidant capacity, with nearly 1.8-fold higher values for all three assays than the general average value of the other genotypes (Figure 1a–c). More specifically, ABTS values ranged between 4.32 to 20.88 mg of trolox equivalant (TE)/g of dry weight (dw) basis (Figure 1a). The ABTS activity of G9 (20.88 mg TE/g dw) did not differ from the corresponding value for the G14 genotype, whereas the rest of genotypes–with the exception of G13 and G16–had values ranging from 8.95–15.96 mg/g. Other studies reported ABTS activity of *C. maritimum* of about 0.43 mg TE/mL [29] and 0.095–0.418 mg/mL, expressed as IC_50_ [14].

In the same way, DPPH ranged from 2.24 to 14.92 mg of TE/g dw in different genotypes of *C. maritimum*, exhibiting the following descending order: G14 > the majority of genotypes > G13 and G16 (Figure 1b). Generally, DPPH values in native Greek genotypes of *C. maritimum* presented an average value of 8.0 mg of TE/g dw, which is in accordance with those of Sousa et al. [30], who found a value of 7.3 mg of TE/g dw. Previous studies in the literature presented a DPPH scavenging activity of *C. maritimum*, expressed as IC_50_, of around 7.6 mg/mL [29], or ranged from 0.15 to 1.21 mg/mL [14].

In agreement with the abovementioned results obtained for ABTS and DPPH were those obtained by the FRAP assay, which presented the same trend (Figure 1c).

The AsA content of native Greek *C. maritimum* showed a mean value of 27.23 μmol/g of dw basis, ranging from 8.83 to 40.70 μmol/g (Figure 1d). Specifically, the genotype G4 from Ikaria Island and G9 from Kefalonia Island had, numerically, the greater AsA content of 40.70 μmol/g, a value that did not differ from the respective values of the other studied genotypes (G12, G14, G15, G17, and G18). However, the AsA content was significantly lower in several native Greek genotypes such as G6, G5, G13, G3, G1, and G16.

**Figure 2:**
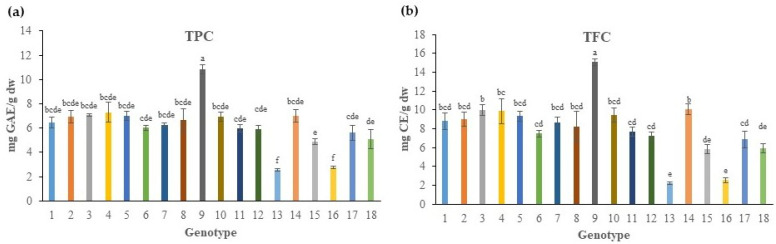
The content of total phenolic compounds (TPC) (**a**), expressed as mg of gallic acid equivalents (GAE) per g of dry weight (dw), and total flavonoid compounds (TPC) (**b**), expressed as mg of catechin equivalents (CE) per g of dw in the extracts of aboveground portion of 18 native Greek rock samphire genotypes. Data represent the mean values ± standard deviation. Different letters on the bars characterize significant differences among genotypes according to Tukey’s test for *p* ≤ 0.05.

An ANOVA on data obtained from the evaluated parameters of bioactive compounds [total phenolic content (TPC) and total flavonoid content (TFC)] showed significant effects due to genotype of 18 native Greek rock samphire as a source of variation (Table 2). The TPC of genotypes ranged from 2.55 to 10.84 mg gallic acid equivalents (GAE)/g of dw basis (Figure 2a). Specifically, genotype G9, derived from Kefalonia Island, had the greater TPC value of 10.84 mg GAE/g dw, followed by the majority of genotypes, with the exception of G13 and the G16, which showed the lowest values for TPC. Generally, the rest of the studied germplasm presented small or ameliorated TPC differences, ranging from 4.89 mg/g in G14 from Heraklion, Crete to 7.30 mg/g in G4 from Ikaria Island. Comparing the results with that found in the literature, a previous report [29] presented the TPC of rock samphire extracts to be about 47 mg GAE/g dw, a value that is eight-fold higher than the mean value of 6.18 mg/g dw found in the present study. Recently, other researchers [31] stated that *C. maritimum* leaves contained 31.7 mg/g dw of TPC, or a similar level of 23–33 mg/g dw depending of the season (spring-summer) [14]. Conversely, Sánchez-Faure et al. [18] found a TPC of rock samphire of about 8.6 mg/g dw, a value closer to the one found in the current study harvested before flowering, in the middle of July. Moreover, another study [32] reported a value of 2.3 mg/g dw for TPC in the aerial parts of rock samphire, a content similar to the lower values found in the present study.

Generally, the TPC content presents a wide variation of 0.5 to 132 mg GAE/g dw among the different halophytes from the Mediterranean area [33], whereas it occurs at a level of about 6 mg/g dw in different vegetables [34]. In the current study, the majority of the tested genotypes of *C. maritimum* had about 6.0–7.3 mg/g dw of TPC, a content slightly higher than that of other vegetables. Recently, Martins-Noguerol et al. [35] found that the TPC content of rock samphire under optimum conditions was 6.1 mg/g dw, even though plants under field conditions exhibited a high phenolic content of 30.2–48.0 mg/g dw regardless of the variability of the contrasting habitats.

Moreover, Zhou and Yu [36] reported that some vegetables, such as spinach and broccoli, had TPC contents of 13 and 10.84 mg/g dw, respectively. Notably, in our results, G9 had a comparable level of TPC to the values reported for spinach and broccoli.

The TFC content of the various genotypes ranged from 2.25 to 15.08 mg of catechin equivalents (CE)/g dw (Figure 2b). Similar to the TPC content, the highest TFC value was observed in G9, followed by the majority of genotypes, while the lowest values were found in G13 and G16 genotypes of *C. maritimum*, which is in line with the TPC results. More precisely, most of the genotypes contained TFC in the 5.82–10.02 CE/g dw range. Rock samphire derived from different climatic areas exhibited contrasting TFC contents. For example, Nabet et al. [29] reported that the TFC content of rock samphire extracts from Algeria was 17 mg/g dw, a value near the maximum of TFC content that we found in G14, whereas another study [32] reported a content of 2.3 mg/g dw of TFC in aerial parts of rock samphire from the Croatian Adriatic coast, a value similar to the lower value found in two native Greek genotypes in the present study. On the other hand, Souid et al. [31] found that *C. maritimum* leaves from the French Atlantic coast contained 25.6 mg/g of TFC. In addition, previous researchers [37] stated that the TFC content of infusions and decoctions of different above-ground organs of rock samphire from the Portugeuse Atlantic coast ranged from 25 (in stems) to 55 (in leaves) mg rutin equivalents/200 mL (a cup of tea).

### 2.2. Identification and Quantification of Phenolic Compounds

**Figure 3:**
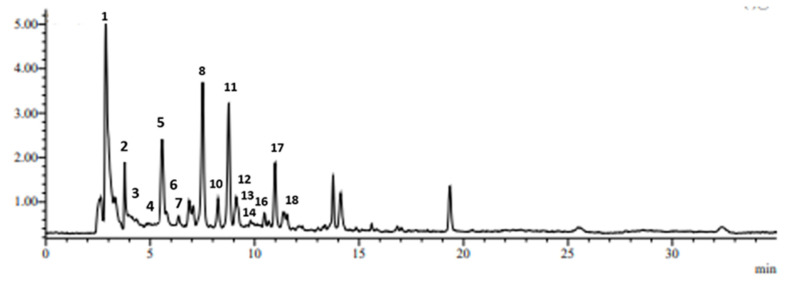
Representative total ion current chromatographic (TIC) profile of rock samphire extract (genotype G16) obtained by HPLC-MS.

The chromatographic profile of the native Greek rock samphire’s extracts identified by liquid chromatography-mass spectrometry (LC-MS) analysis is shown in Figure 3. The LC-MS analysis allowed the identification of 18 main phenolic compounds, which were separated and characterized regarding their retention time, UV, and MS spectra data (Table 3). Eleven of them belong mainly to quinic acid derivatives (peaks 1, 2, 3, 5, 6, 8, 10, 11, 16, 17, and 18), four of them are characterized as flavones (peaks 7, 12, 13, and 14)–mainly quercetin derivatives–, two of them are classified as hydroxycinnamic acids (peaks 9 and 15), and one of them as hydroxybenzoic acid (peak 4). All of the detected peaks were identified by the use of reference standards; however, the peaks 8, 10, and 11 were identified based on data from the literature. Four peaks (peaks 2, 3, 5, and 6) appeared at 4.01, 4.34, 5.65, and 5.85 min, respectively, with pseudo-molecular ion [M-H]^−^ at m/z = 353 and UV maximum at 325 nm corresponds to 1-caffeoyl-quinic acid (1-CQA), 5-O-caffeoylquinic acid (5-CQA or neochlorogenic acid), 3-O-caffeoylquinic acid (3-CQA or chlorogenic acid), and 4-O-caffeoylquinic acid (4-CQA or cryptochlorogenic acid), respectively. The peaks 16, 17, and 18 that appeared at 10.45, 10.95, and 10.61 min, respectively, with pseudo-molecular ion [M-H]^−^ at m/z = 515 and UV maximum at 327 nm were assigned to dicaffeoyl quinic acids as 3,4-dicaffeoyl-quinic acid (3,4-DCQA), 3,5-dicaffeoyl-quinic acid (3,5-DCQA), and 4,5-dicaffeoyl quinic acid (4,5-DCQA), respectively. Four peaks appearing at 6.36, 9.21, 9.63, and 9.78 min were characterized as flavonoids, e.g., vicenin-2 (VIC), quercetin-3-O-rutinoside (QURU or rutin), quercetin-3-O-glucoside (QUGL), and quercetin-3-O-galactoside (QUGA or hyperoside), respectively. Three minor peaks detected at 5.01, 7.61, and 9.98 min presenting [M-H]^−^ at m/z = 137, 179 and 163 were identified as protocatechuic acid (PRCA), caffeic acid (CA), and *p*-coumaric acid (pCA), respectively.

The peaks 8 and 11 at 7.51 and 8.84 min, respectively, showing pseudo-molecular ion [M-H]^−^ at m/z = 337 and UV maximum at 311 nm were attributed to a coumaroyl quinic acid isomers (5-cQA) [29,38]. Similarly, peak 10 detected at 8.24 min having UV maxima at 297 and 325 nm and pseudo-molecular ion [M-H]^−^ at m/z 367 was attributed to a feruloyl quinic acid (5-fQA) derivative.

An ANOVA applied to the 18 major phenolic compounds derived from 18 native Greek rock samphire genotypes revealed significant differences among genotypes (Table 2). Generally, caffeoylquinic acids (CQAs) were the major phenolic compounds quantified by LC-MS, ranging from 414 to 1547.3 mg/100 g dw, with a mean value of 1089.4 mg/100 g dw (Table 4). The predominant CQAs quantified were 5-cQA, followed by 3-CQA (or CLA), 3,5-DCQA, 1-CQA, and 5-fQA, presenting mean values of 369.2 (sum of isomers/peaks 8 and 11), 258.7, 211.1, 109.3, and 74.6 mg/100 g dw, respectively (Table 4).

Other minor CQAs quantified in rock samphire’s genotypes were 4-CQA (cryptochlorogenic acid), 5-CQA (neochlorogenic acid), 3,4-DCQA, and 4,5-DCQA, with mean values of 21.4, 18.3, 15.1, and 11.9 mg/100 g dw, respectively. Regarding the flavonoid class, QURU and VIC were the major compounds detected (20.3 and 14.2 mg/100 g dw, respectively), along with QUGL and QUGA as the most abundant flavone derivatives (Table 5). Finally, among other phenolic acids (OPAs), which are summarized in Table 6, only QNA was found with 82.8 mg/100 g dw, whereas the concentrations of PRCA, CA, and pCA was negligible. Concerning the rock samphire’s phenolics identification by LC-MS, our results resemble those of other researchers [39], who identified three caffeoylquinic acid isomers (3-CQA, 4-CQA, and 5-CQA) and three dicaffeoylquinic acids (3,4-DCQA, 3,5-DCQA, and 4,5-DCQA), which were also presented in the current work.

It should be noted that, as mentioned above, 5-CQA, 3-CQA, and 4-CQA are the neochlorogenic, chlorogenic, and cryptochlorogenic acid, respectively, whereas, generally, quinic acid is a component of a chlorogenic acid moiety [9]. According to previous research [9], rock samphire is among the richest phenolic-containing species within the Apiaceae family, with caffeoylquinic acid derivatives being the sole class of phenolics [40]. In agreement with our results, Mekinić et al. [41] reported that 3-CQA ranged from 5.65 to 7.48 mg/g dw, while it was 16.28 mg/g dw at an April harvest.

Moreover, a previous study [1] reported that, among seventeen constituents identified, the main phenolics were 3-CQA, 4-CQA, 5-CQA, and 1.5-dicaffeoylquinic acid. Our results are in accordance with the findings of that study [1] concerning the concentrations of 4-CQA and 5-CQA; nevertheless, they are in contrast in the case of 3-CQA and 1-CQA. More specifically, we found over fifteen-fold more 3-CQA and over two-fold more 1-CQA concentrations (averaged over genotype) compared with the respective values reported by Sarrou et al. [1]. These differences may be partially attributed to the different initial material used in the two studies, as, in the current work, the whole edible above-ground part was used at a ratio of leaves:stems 1:1 (w:w), whereas in Sarrou et al. [1], a ratio of 2:1 was used. Moreover, our results are comparable with those of Nabet et al. [29], who revealed that hydroxycinnamic acids were among the main phenolic compounds of rock samphire from Algeria according the following order of importance: 5-CQA (636 mg/100 g dw) > 3,5-DCQA (164 mg/100 g dw) > 5-cQA and 5-fQA (104 mg/100 g dw) > 1-CQA and 4,5-DCQA (each one of 103 mg/100 g dw). In the current work, 5-CQA was more than thirty times lower (18.26 mg/100 g dw), but 5-cQA content was about three times higher (369 mg/100 g dw) than in those results [29]. Concerning the 3-CQA (chlorogenic acid) content, other researchers [9] found huge concentrations of 1880–2790 mg/100 g dw in rock samphire growing in sand and 300–1000 mg/100 g dw in plants growing on cliffs, compared to the 258.73 mg/100 g dw found in the current study.

Genotypes of native Greek rock samphire were rich in quinic and chlorogenic acids, despite the fact that halophytic species are usually pure in these compounds. However, quinate and/or chlorogenate were detected in several members of the Apiaceae family, including carrot [42,43], fennel [44], and celery [45]. Based on the aforementioned knowledge, Meot Duros and Magné [20] stated that these two molecules could play a chemotaxonomic role in the Apiaceae family. In any case, rock samphire, similar to fennel and greater than carrot, can be considered among the 3-CQA (or CLA)-richest Apiaceae members, with concentrations up to 259 mg/100 g dw [44].

Contrary to a previous study [13], other phenolic compounds such as catechins, vanillic, and rosmarinic acids were undetectable in the present work. Nevertheless, it should be not disregarded that differences in the ranges of estimated parameters among the results of the current study and similar studies could be attributed to factors such as climatic conditions, harvesting time, and extraction method (solvent, temperature, time, etc.).

Altogether, the eighteen components of native Greek rock samphire’s extracts had a total polyphenol content of 1214.4 mg/100 g dw, a half-reduced value compared with the 2503 mg/100 g dw reported by a previous study [31].

Regarding the differences among genotypes, the G9 genotype from Kefalonia Island had cumulatively CQAs of 1547.3 mg/100 g dw, followed by G1-G5, G7, G8, and G10, while G13 and G16 had the lowest content (Table 4). It is remarkable that the genotypes G1, G3, G4, and G5 exhibited high CQAs values originating from the same island, namely Ikaria. Concerning the major quinic acids, it seems that G9, G8, and G2 contained more 5-cQA than the others, whereas G9 and G3 had more 3,5-DCQA (Table 4). Similarly, G9 had the highest 1-CQA concentration, although not statistically different than the respective values of several other genotypes (such as G2–G5, G7, and G10). With regard to chlorogenic acids, the G9 genotype had the highest content, even though the majority of genotypes had also high contents (Table 4). In addition, G9, G2, and G4 had the highest total concentration of FLAs (Table 5).

### 2.3. Correlation Coefficients among Bioactive Compounds, Antioxidant Capacity, and Phenolic Groups

The correlation Pearson coefficient (r) analysis was used to evaluate the relationship between bioactive compounds, antioxidant capacity, and phenolic groups. Significant positive correlations (*p* ≤ 0.001) were observed between the vast majority of traits (Table 7), which indicated that when selection or breeding are applied for one of these traits, an indirect improvement could also be observed in other traits [46]. Clearly, TPC was strongly correlated with TFC, ABTS, DPPH, FRAP, CQAs, and FLAs (r = 0.65–0.99, *p* ≤ 0.001), but it had no significant correlation with OPAs (r = 0.102, *p* > 0.05) and no correlation with AsA (r = 0.332, *p* > 0.05).

Similarly, TFC was highly correlated with all the other traits (r = 0.633–0.964, *p* ≤ 0.001), except from the case of OPAs and AsA, where no significant correlation was observed (r = 0.141, *p* > 0.05 and r = 0.317, *p* > 0.05, respectively). ABTS was very strongly correlated with DPPH, FRAP, and CQAs (r = 0.815–0.882, *p* ≤ 0.001), highly correlated with FLAs (r = 0.559 *p* ≤ 0.001), but it was not correlated with OPAs (r = 0.066, *p* > 0.05) and AsA (r = 0.420, *p* > 0.05). The same trend was observed for the rest of the traits, except for the cases of OPAs and AsA, where no correlation with any other trait was detected.

These findings clearly show that TPC and TFC contribute, to a high extent, to the antioxidant activity of rock samphire’s native Greek genotypes. Moreover, the phenolic groups were identified successfully by LC-MS in the current study and they corresponded, to a great extent, to the determined TPC and TFC, as well as to antioxidant capacity, whereas our findings are in agreement with earlier reports [47,48]. Indeed, Xu et al. [47] reported that CQA isomers (specifically 3-CQA, 4-CQA, and 5-CQA) and DCQA isomers (specifically 3,5-DCQA, 3,4-DCQA, and 4,5-DCQA) exhibit antioxidant activities and DNA damage protective effects to various extents. In the same direction, Kooti et al. [48] have reported that the major phenolic compounds, such as CQAs, of fennel seed extracts show important antioxidant activity.

### 2.4. Principal Component Analysis and Hierarchical Clustering

**Figure 4:**
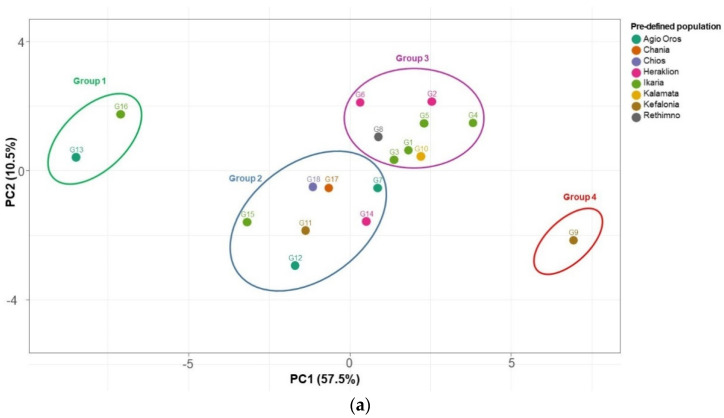

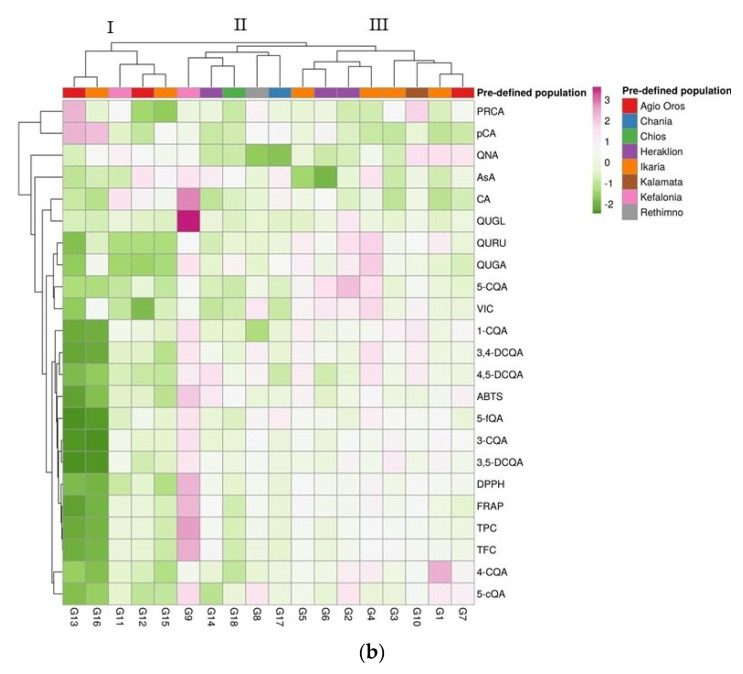
Two-D PCA plot of the first two components of 18 native Greek rock samphire genotypes (see Table 1) based on 23 determined traits related to bioactive compounds and antioxidant activity (**a**), and a heat map showing fold changes of the same traits of the 18 rock samphire genotypes (**b**). Columns are centered; unit variance scaling is applied to columns. Columns are clustered using Euclidean distance and Ward linkage (23 rows, 18 columns).

In total, 23 determined variables (3 bioactive compounds, 3 antioxidant capacity assays, and 17 phenolic compounds identified by LC-MS) of 18 rock samphire genotypes were subjected to a principal component analysis (PCA). Based on an eigenvalue > 1, we extracted a total of two PCs with a cumulative distribution of 68.0%, (specifically 57.5% for the first component and 10.5% for the second one) (Figure 4a). Generally, native Greek rock samphire genotypes formed four distinct groups, where the majority of genotypes were placed in the center of the PCA, mainly in the lower left (group 2) and in the upper right quarter (group 3). Interestingly, G9 from Kefalonia Island was identified alone and it was located in group 4, close to the positive side of PC1, presenting a rather similar response with high values at most evaluated parameters. Additionally, the genotypes G5, G4, G3, and G1, all originating from Ikaria Island, were included in group 3 and they were ordinated to the positive side of PC1 and PC2 in the upper right quadrant, indicating a tendency for high values in most evaluated traits. Notably, among them, G4 from Ikaria Island was separated from the rest by the higher positive loadings on PC1. Conversely, the genotypes of group 2 presented values of phytochemical and antioxidant capacity below the mean value, and were thus grouped at the lower left quarter. Moreover, G13 and G16 genotypes–the only members of group 1 lined with the green color in Figure 4 and located at the left side–presented the most distinct and significant reduced values for the evaluated parameters.

Stepping forward to investigate the differences on rock samphire genotypes, an agglomerative hierarchical clustering (AHC, heatmap) analysis on the bioactive content, antioxidant capacity, and main phenolics was employed to enable the grouping of genotypes into clusters of similar responses based on calculations of the Euclidean distance (Figure 4b). The resulting dendrogram using the Ward’s method for agglomeration, revealed three distinct groups: Group I and Group II, which were comprised of five genotypes each, and Group III, which contained eight genotypes. More specifically, the heatmap analysis identified a subgroup of genotypes G13 and G16 within Cluster I based on the lower values in the vast majority of traits. The genotypes G11, G12, and G15 were grouped in the same Cluster I. A subgroup of Cluster II contained only the G9 genotype from Kefalonia Island, exhibiting high values for most of the parameters, which is highlighted with a strong pink color in Figure 4b. The same Cluster II contained the genotypes G14, G8, and G17 from Heraklion, Rethimno, and Chania (Crete), respectively, and G18 from Chios Island. Additionally, Cluster III presented fluctuating responses in different estimated variables, whereas half of the included genotypes originated from Ikaria Island. Specifically, a subgroup consisting of G2 and G6 from Heraklion and G5 and G4 from Ikaria presented medium to high values in most estimated traits. Notably, G4 from Ikaria presented impressively high values on most estimated variables and is thus indicated with different shades of pink color in Figure 4b. A different subgroup of Group III, comprised of the G1, G3, G9, and G7 genotypes, presented small fluctuations around the mean values of estimated variables. Finally, results from the AHC are in accordance with the PCA.

### 2.5. Genetic Diversity and Molecular Characterization

**Figure 5:**
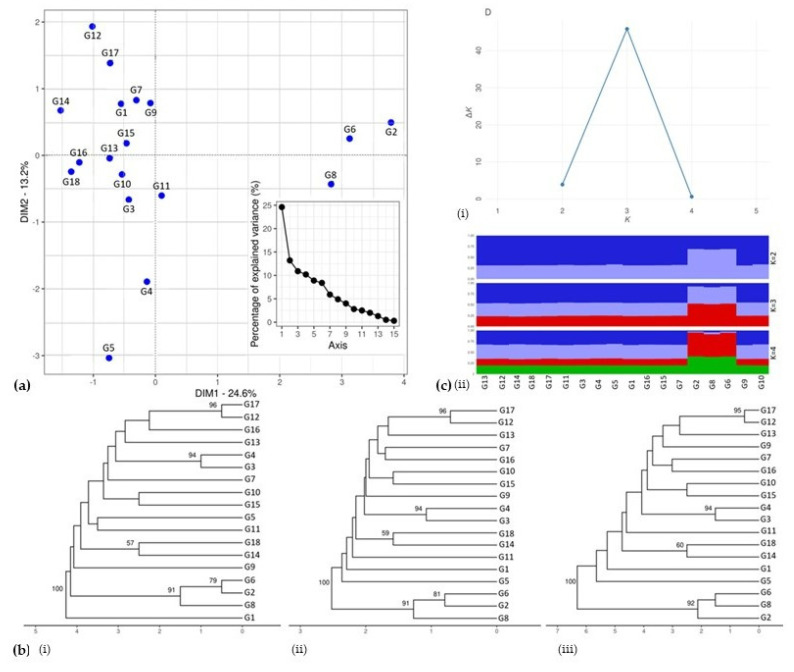
(**a**) Principal Coordinate Analysis (PCoA) plot of 18 native Greek rock samphire genotypes (Table 1) with the percentage of the explained variance (%) demonstrated on the right side of the figure. (**b**) UPGMA dendrogram computed by using three distance measures, i.e., (**i**) Relative dissimilarity matrix, (ii) Euclidean Distance, and (**iii**) Smouse and Peakall distance. (**c**) Distribution of 18 rock samphire genotypes according to molecular data of 8 SSR loci in the Structure software with (**i**) values of Evanno’s ΔK statistic indicating the most probable genetic structure model and (**ii**) genetic assignment based on STRUCTURE. The individuals are represented by vertical bars; the colors were assigned according to the group formed in the Structure software (three groups, K = 3).

Concerning the GenAlEx analysis for allelic patterns of the examined samples from *C. maritimum* native Greek populations, i.e., Agio Oros (3 genotypes), Chania (1), Chios (1), Heraklion (3), Ikaria (6), Kalamata (1), Kefalonia (2), and Rethimno (1), the results showed that the mean number of different alleles (Na) was 5.500, while the mean number of different alleles with a frequency ≥5% was 4.375. Moreover, the number of effective alleles (Ne) was 3.131, Shanon’s information Index (I) was 1.181, and the unbiased expected heterozygosity (uHe) was 0.577 (Table 8). The combined probability of identity (PI) was 1.5 × 10^−6^, while the combined value for PIsibs was 4.8 × 10^−3^. The results, according to a PCoA analysis, classified the genotypes into three groups. Most of the genotypes were concentrated on the left side of the plot. G2, G6, and G8 formed another group in the right part of the plot. A third cluster is detected in the bottom left quadrant, consisting of G4 and G5. According to the percentage of explained variance (%), which is used to measure the discrepancy between a model and actual data, a small variance between genotypes was revealed, while the percentage of this variance was below 60% (Figure 5a).

The three unweighted pair group method with arithmetic average (UPGMA) dendrograms were in concordance with each other, with two main clusters appearing in all of them. According to the first dendrogram using the relative dissimilarity matrix, a large cluster included the majority of genotypes, while a second cluster consisted of G2, G6, and G8. G1 was clustered separately from all the other genotypes [Figure 5(bi)]. The following two dendrograms using the Euclidean Distance and the genetic distance of Smouse and Peakall, respectively, seemed to support the same pattern, forming two clusters with the main one comprising most genotypes and the second one including three genotypes (G2, G8, and G6) [Figure 5(bii,biii)].

To gain further insight, a STRUCTURE analysis was also conducted. In this analysis, K = 3 was deemed as optimal according to the ΔK statistic of Evanno [49]. However, K = 2 also produced a solution that could have biological relevance. For K = 3, the first group assembled all accessions, except from genotypes G2, G8, and G6 which formed a second cluster [Figure 5(ci)]. A similar result was obtained for K = 2 in concordance with the corresponding dendrogram [Figure 5(cii)].

Lately, there has been an escalation in phylogeographical studies regarding coastal and halophytic plants, not only in the Mediterranean Basin, but also along the European coasts [50]. Numerous hypotheses have been suggested to explain the present genetic structure and natural distribution of halophytic species. Understanding the evolutionary history of populations that have emerged from various complex events is challenging work. Hence, researchers should be able to examine different molecular markers from different genomes to comprehend the differences in evolution between species [51]. In contrast to other types of molecular markers, microsatellites, or simple sequence repeats (SSRs), have many advantages, such as simplicity, effectiveness, abundance, hypervariability, reproducibility, co–dominant inheritance, and extensive genomic coverage [52]. The SSRs markers have become one of the most useful molecular markers for cultivar fingerprinting and genetic diversity assessments, molecular mapping, positional cloning, phylogenetic analyses, and marker-assisted breeding [53]. They have successfully been used for many plant species, such as rice (*Oryza sativa*), maize (*Zea mays*), barley (*Hordeum vulgare*), and sorghum (*Sorghum bicolor*) [54,55,56,57].

Halophytic plants are ideal models for studying the colonization routes of species because of their simple linear distributions across the coastlines. Thus, a couple of studies have been carried out using SSR molecular markers to analyze the phylogenetic relationships between species and populations of halophytes. In 2010, Escudero et al. [58], made a successful attempt to examine the evolutionary history of 24 populations of the halophyte *Carex extensa*, analyzing its genetic structure with the aim of understanding the heterogeneity of closely related halophytic species. The results indicated that *C. extensa*, together with the South American *Carex vixdentata* and the southern African *Carex ecklonii*, form a monophyletic group of halophytic species [58]. Another study [59] investigated *Nitraria sibirica*’s chloroplast genome to provide insights into comparative genome analysis, and to understand the phylogenetic relationships within the Sapindales. This evolutionary analysis showed that *N. sibirica* belongs to the order Sapindales, providing valuable information about halophytes in general [59]. Evolutionary studies have been performed at the genome level to untangle the conserved features of *Oryza coarctata* within the genus *Oryza*, and to uncover its similarity with other halophytic species and differences from the AA, BB, and FF genome types present in members of the genus *Oryza* [60]. Further phylogenetic analysis, based on the single copy genes among *Oryza* species, pointed to the existence of the *O. coarctata* genome somewhere between the divergence of the FF and BB genomes from the AA genome [60]. In addition, another study identified a number of genic SSR markers from the transcriptomic dataset, providing valuable resources for future ecological and evolutionary studies of *Phragmites karka* [61]. Regarding *C. maritinum*, few studies have been performed to date to investigate and comprehend the phylogenetic relationships between closely related species and populations. Two studies presented in 2005 using an Amplified Fragment Length Polymorphism (AFLP) marker have shown several incipient geographical lineages in the Mediterranean and European coastal regions for *C. maritimum*, but without any noteworthy support, indicating, however, that its colonization happened after the Last Glacial Maximum [21,62].

The results herein indicated a conformity between the analyses from GenAlEx, UPGMA, and STUCTURE software. As evident from the PCoA plot, most native Greek genotypes have similarities to each other, forming a group on the left part of the plot, comprised of genotypes from Kalamata, Chania, Agio Oros, Kefalonia, Ikaria, Chios, and Heraklion. A little further away from these genotypes, there are G2 and G6, 2 genotypes from Heraklion, and G8 from Rethimno. According to the GenAlEx analysis, the results showed low to moderate diversity between native Greek populations, while the expected heterozygosity (He) was 0.560, and the unbiased expected heterozygosity (uHe) was 0.577. Moreover, Shanon’s information Index was quite low in the amount of 1.131. The number of different alleles was 5.500 and the number of effective alleles was 3.131, also pointing to a low diversity pattern. In addition, the low levels of the percentage of the explained variance (under 60%) also indicate the low variability detected between Greek populations of *C. maritimum*.

A genetic structure of a population can generally be interpreted by the amount and distribution of genetic variation within and between populations. According to STUCTURE software, one main group is formed comprised of most of the genotypes and excluding only three of them (G2, G6, and G8) from Heraklion and Rethimno. Therefore, a resemblance between genotypes can be inferred, without significant divergence.

The method of unweighted average binding among clusters, better known as UPGMA, has been used most frequently in ecology and systematics [63], as well as in numerical taxonomy [64]. Regarding the results obtained in this study, we can notice once more the two formed groups on the dendrograms below, which are in agreement with the PCoA and STRUCTURE analysis, excluding genotypes G2, G6, and G8 from Heraklion and Rethimno.

Genetic diversity is crucial for a population to allow for adaptation to changing environments. Gene flow within a population could increase genetic variation, with numerous factors affecting it. Gene duplication, mutation, or other physical processes are some factors leading to this increment. In addition, new variations could be created when a population has high reproduction levels. On the other hand, small populations are more likely to undergo diversity loss gradually, by random chance, via genetic drift. Factors that cause genetic drift might be the unlike number of offspring by different members of a population, such that specific genes increase or decrease in number over generations free of selection, the unexpected immigration or emigration of individuals, resulting in changing genes, etc. Besides genetic drift, gene flow is supposed to be lower in species that have a small distribution range or low adaptability, that arise in fragmented environments, where populations are geographically distant, and the population sizes are small. Overall, it is argued in this study that many island populations of *C. maritimum* in Greece might have low rates of gene flow and thus suffer high genetic drift due to geographic isolation and small population sizes, with the latter being so isolated that the lack of gene flow may lead to high rates of inbreeding.

## 3. Materials and Methods

### 3.1. Reagents

Analytical standards of quinic acid (QNA), 3-O-caffeoylquinic acid (3-CQA), 4-O-caffeoylquinic acid (4-CQA), 5-O-caffeoylquinic acid (5-CQA), quercetin-3-O-rutinoside (QURU), quercetin-3-O-glucoside (QUGL), and quercetin-3-O-galactoside (QUGA) were purchased from Extrasynthese (Genay Cedex, France), whereas protocatechuic acid (PRCA), gallic acid (GA), caffeic acid (CA), and *p*-coumaric acid (pCA) were obtained from Sigma-Aldrich (Steinheim, Germany). 1-caffeoylquinic acid (1-CQA), 3,5-dicaffeoylquinic acid (3,5-DCQA), 3,4-dicaffeoylquinic acid (3,4-DCQA), 4,5-dicaffeoylquinic acid (4,5-DCQA), and vicenin-2 were obtained from Carbosynth (Berkshire, UK). All other reagents were of HPLC or LC-MS grade.

### 3.2. Plant Material

**Figure 6:**
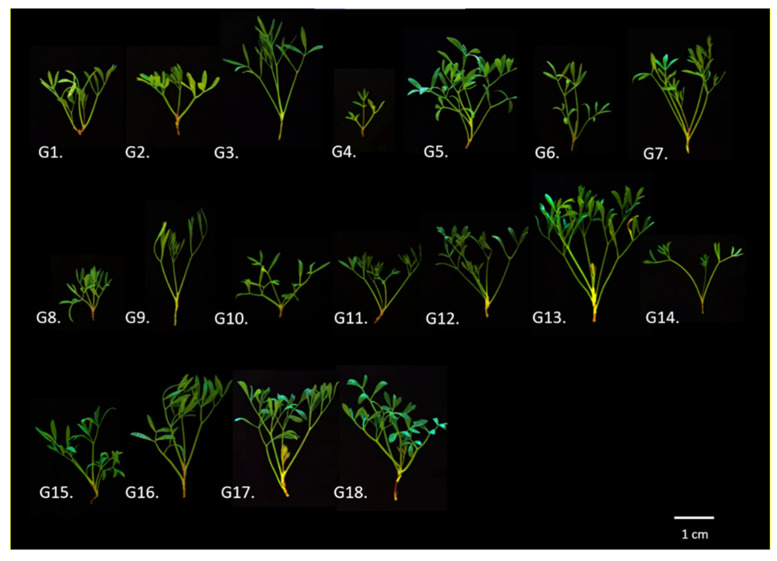
Indicative visual phenotype of the 18 native Greek rock samphire genotypes studied. Explanations for the abbreviations G1–G18 are given in Table 1.

The plant material used in this study is part of the *C. maritimum* germplasm collection maintained ex situ at the grounds of the Balkan Botanic Garden of Kroussia in Northern Greece (Pontokerasia: 41°05′24″ N, 23°06′43″ E and Thermi: 40°32′08.7″ N, 23°00′06.4″ E). All plant materials of *C. maritimum* have been collected directly from wild-growing populations based on sustainable plant exploitation strategies. The collections were conducted using a special permit to the Institute of Plant Breeding and Phytogenetic Resources, Hellenic Agricultural Organization–Dimitra issued by the Greek Ministry of Environment and Energy, which is renewed annually after a detailed report (for the last two years Permit 82336/879 of 18 May 2019 & 26895/1527 of 21 April 2021). Each genotype after taxonomic identification is allocated a unique IPEN (International Plant Exchange Network) accession number given by the Balkan Botanic Garden of Kroussia (BBGK), Institute of Plant Breeding and Genetic Resources (IPB & GR), Hellenic Agricultural Organization-Dimitra. A total of 18 rock samphire accessions with geographic origin from 8 different and representative regions of Greece were selected for further study and they are presented with relative information in Table 1 and Figure 6.

### 3.3. Sample Preparation

From each of the rock samphire genotypes grown under the same ex situ conditions, 500 g of fresh aerial parts was collected in the stage before flowering in the middle of July, when the plants reached their maximum foliage. Samples were collected and put in sterile polyethylene carrier bags and transported to the laboratory in a portable refrigerator (3–4 °C) not more than one hour after their collection. The fresh samples were divided into leaves and stems and then they were weighed on a digital balance with an accuracy to 0.01 g to calculate their ratio and to avoid tissue-specific differential content in samples. Separated tissue samples were put in plastic bags and were freeze-dried for 72 h with a lyophilizer (Freeze-dryer Alpha 1–2 LD plus, Christ, Osterode, Germany) until they obtained their dw. Afterwards, samples were grounded in a laboratory mill (ZM 1000, Retsch GmbH, Haan, Germany) to pass through a 0.50 mm sieve, and then they were stored at −20 °C until analysis.

### 3.4. Evaluated Parameters

Several indicators regarding the antioxidant potential of *C. maritimum* (expressed as ABTS and DPPH radical scavenging activity, as well as a FRAP assay), the AsA content, and the bioactive profile (as TPC and TFC) were determined, along with the main phenolic compounds identified and quantified by LC-MS (all described below).

#### 3.4.1. Sample Extraction

To extract each sample, 400 mg of freeze-dried and powdered sample derived of equal portions of leaves and stems of *C. maritimum* were transferred to glass vials containing 4.5 mL of methanol/water (70:30, v/v). The suspension was vortexed for 1 min and then incubated in an ultrasound bath (model FB15051, Thermo Fisher Scientific Inc. Loughborough, UK) for 20 min. Afterwards, the crude extract was centrifuged at 4000× *g* for 10 min, the supernatant was collected, and the pellet was re-extracted as described above. Each extraction was triplicated and all analyses were performed in three replications.

#### 3.4.2. Antioxidant Capacity Determination

For a valid assessment of antioxidant capacity, a combination of methods was important [65]. For this reason, the antioxidant capacity was determined according ABTS and DPPH reactive oxygen species scavenging assays and a FRAP redox potential-based assay. Specifically, ABTS and DPPH scavenging assays are based on electron donation of antioxidants to neutralize ABTS and DPPH radical cations, whereas the FRAP assay is a typical, single electron transfer-based method that measures the reduction of a ferric ion (Fe^3+^)–ligand complex to the intensely blue-colored ferrous (Fe^2+^) complex by antioxidants in acidic media.

*ABTS Radical Scavenging Activity*

The radical scavenging activity of rock samphire’s extracts against the ABTS radical cation was evaluated according to the protocol of Re et al. [66], which was appropriately adjusted. Briefly, 100 μL of the sample extract was added to 3.9 mL of diluted ABTS^+^ solution and the absorbance was measured at 734 nm after 4 min against a blank (methanol). The results were expressed as mg trolox equivalents (TE) per g of dried sample (mg TE/g dw).

*DPPH Radical Scavenging Activity*

The DPPH radical scavenging activity of rock samphire’s extracts against the DPPH radical cation was evaluated according to the protocol of Yen and Chen [67], with some modifications. Briefly, 150 mL of the extract was reacted with 2.85 mL of a 0.1 mM methanolic solution of DPPH. After 5 min, the absorbance at 516 nm was recorded, with methanol being used as the blank. The percentage of scavenging effect was calculated by using the following equation: DPPH radical scavenging capacity (%) = (A_0_ − A_s_)/A_0_ × 100, where A_0_ and A_s_ are the absorbance of the blank and the sample, respectively. Results were expressed as mg of TE per g of dried sample (mg TE/g dw).

*FRAP Assay*

The FRAP assay was carried out according to Benzie and Strain [68], with slight modifications. Briefly, the fresh FRAP reagent consisted of 20 mM ferric chloride solution, 10 mM TPTZ (2,4,6-tripyridyl-s-triazine) solution in 40 mM HCl, and 0.3 mM acetate buffer pH 3.6 in a proportion of 1:1:10, respectively. An aliquot of 100 μL of sample extract was reacted with 3 mL of the FRAP reagent at 37 °C for 4 min under dark conditions, and the absorbance was recorded at 593 nm against a blank (methanol). Results were expressed as mg of TE per g of dried sample (mg TE/g dw).

#### 3.4.3. Ascorbic Acid Determination

The AsA content was determined spectrophotometrically using the ascorbate oxidase (AO) enzyme as previously described [69]. Briefly, 1 g of frozen tissue (*C. maritimum* stems and leaves, 1:1) were ground to a powder using liquid nitrogen, and were extracted using 1 M HClO_4_. After centrifugation, supernatants were neutralized to pH 5.6 using K_2_CO_3_. Calculations were based upon the difference in absorbance at 265 nm before, and 3 min after the addition of 1 U/μL AO (Sigma Chemical Co., St Louis, MO, USA) to a 200 μL aliquot of extract in 200 mM sodium phosphate buffer (pH 5.6). Absorbance values were evaluated against a standard curve constructed using L-ascorbic acid (Merck KgaA, Darmstadt, Germany) in the range of 0–100 nmol and expressed as μmol/g of dw.

#### 3.4.4. TPC Determination

The analyses of TPC were performed using the Folin–Ciocalteu’s method according to Singleton et al. [70], with some modifications. Briefly, 0.2 mL of sample extract was mixed with 0.8 mL of the Folin–Ciocalteu reagent. After incubation for 2 min, 2 mL of sodium carbonate (7.5% w/v) solution was added to the reaction mixture and the volume was adjusted to 10 mL with distilled water. The mixture was allowed to stand for 60 min in a dark place, and then the absorbance at 725 nm was recorded [71]. The results were expressed as mg of gallic acid equivalent (GAE) per g of sample on a dw basis (mg GAE/g dw).

#### 3.4.5. TFC Determination

The TFC of the sample extracts obtained as described above were evaluated by the AlCl_3_ reagent method of Bao et al. [72], with slight modifications. An aliquot of 0.3 mL of extract was pipetted into a test tube containing 2 mL of distilled H_2_O and mixed with 0.225 mL of 5% NaNO_2_. After 5 min, 0.225 mL of 10% AlCl_3_·6H_2_O solution was added, the mixture was allowed to stand for another 5 min, and then 0.750 mL of 2 M NaOH was added. The reaction solution was well mixed, kept for 30 min in the dark, and the absorbance was determined at 510 nm. The results were expressed as mg of catechin equivalents (CE) per g of sample on a dw basis (mg CE/g dw).

#### 3.4.6. LC-MS Identification and Quantitation of Metabolomics (Phenolic Compounds)

Identification and quantitation of the phenolic profile from rock samphire extracts was performed according Irakli et al. [46] on a Shimadzu Nexera HPLC system (Kyoto, Japan) consisting of two pumps, a degasser, a column oven, an auto injector, a diode array detector (DAD), and a single quadrupole mass spectrometer combined with an electrospray ionization (ESI) interface. A Poroshell 120 EC-C_18_ column (4.6 × 150 mm, 4 μm) was used for the separation of phenolic compounds and was thermostated at 35 °C, and the flow rate was set at 800 µL/min. Each extract was filtered (pore size 0.2 μm), then an aliquot of 10 µL was injected while the gradient was 0–5 min (15–25% B), 5–10 min (25–35% B), 10–28 min (35–60% B), 28–28.01 min (60–15% B), and an isocratic elution until 35 min. Mobile phase A was 0.1% formic acid in water and mobile phase B was acetonitrile.

The DAD acquisition ranged from 190 to 400 nm. Τhe mass spectrometer was equipped with an ESI source recorded on a negative ionization mode under the following conditions: interface voltage, +4.5 kV; curved desolvation line (CDL) voltage, 20 V; nebulizing gas (nitrogen) flow, 1.5 L/min; drying gas flow, 15 L/min; block heater temperature, 200 °C; CDL temperature, 250 °C. Mass acquisitions were performed in full scan mode (100–1000 m/z) and selective ion monitoring mode (SIM). Data acquisition and processing was done using Lab Solutions LC-MS software (Shimadzu, Kyoto, Japan).

The identification of all constituents was performed by LC-DAD-MS analysis by comparing the retention time, as well as the UV and MS spectra of the peaks in the extracts with those of authentic reference samples. The method of internal standard (salicylic acid) was applied to quantify the phenolic compounds of *C. maritimum*. Quantification was carried out at SIM mode, constructing calibrations curves of corresponding standard solutions at five concentration levels within the linear range of 0.01 to 4 μg/mL. Correlation coefficients (r^2^) from calibration curves for all the compounds were between 0.9969 and 0.9999. The limit of detection was in the range of 0.007–0.093 ng/mL and the limit of quantification was from 0.021 to 0.282 μg/mL The results of intra-day and inter-day precision was less than 6.1% and 10.6%, respectively. The quantification of coumaroyl-quinic and feruoyl-quinic acids was based on standard curves generated by the 3-CQA due to the lack of commercial standards. Analyses were carried out in triplicate and the results were expressed as mg per 100 g of sample.

### 3.5. DNA Isolation

Isolation of DNA from leaves was performed with the NucleoSpin Plant kit (Macherey–Nagel, Düren, Germany), according to the manufacturer’s instructions. DNA quantity (concentration) was estimated in a NanoDrop™ One/OneC Microvolume UV-Vis Spectrophotometer, by Thermo Fisher Scientific, by determining the absorbance at 260 nm, while DNA quality was determined by 1% agarose gel electrophoresis using MIDORI Green Direct by Nippon Genetics. Samples were then diluted to 25 ng/μL to form working solutions.

### 3.6. Multiplex Polymerase Chain Reaction (PCR) and Capillary Electrophoresis (CE)

Samples were genotyped with eight simple sequence repeat (SSRs) markers (CM03, CM04, CM11, CM12, CM14, CM15, CM33, and CM34). The forward primers were labeled with FAM, HEX, and ROX fluorescent dyes. Multiplex PCR was performed using KAPA2G Fast Multiplex PCR Kit (KAPA Biosystems, Wilmington, MA, USA)). The 2× Multiplex Mix contains KAPA2G Fast HotStart DNA Polymerase (1 U per 25 µL reaction), KAPA2G Buffer A (1.5× at 1×), dNTPs (0.2 mM each dNTP at 1×), MgCl2 (3.0 mM at 1×), and stabilizers. Amplifications were performed in a reaction volume of 25 μL containing 2× KAPA2G Fast Multiplex Mix (12.5 μL), 10 μM Forward Primer (0.5 μL each), 10 μM Reverse Primer (0.5 μL each), DNA (0.5 μL), and PCR-grade Water (up to 25 μL). The PCR was performed in the following thermocycling conditions: 1 cycle of initial denaturation at 95 °C for 3 min, followed by 35 cycles of denaturation at 95 °C for 15 s, annealing at 60 °C for 30 s, extension at 72 °C for 30 s, and 1 cycle of final extension at 72 °C for 1 min. The resulting PCR products were first visualized by 2% agarose gel electrophoresis and then loaded into an SeqStudio Genetic Analyzer, a fluorescence-based capillary electrophoresis system, for fragment analysis (Applied Biosystems, Foster City, CA, USA). SSR fragments were scored via GeneMapper v6 software using the internal size standard GS 600 LIZ (Applied Biosystems, Foster City, CA, USA).

### 3.7. Data Analysis and Cluster Analysis

ANOVA was carried out using the computer software MSTAT-C version 1.41 (Michigan State University, East Lansing, MI, USA). Data of TPC, TFC, ABTS, and DPPH radical scavenging activity, as well as of the FRAP assay, were subjected to an ANOVA by using the experiment model number 7 of one factor (rock samphire genotype) randomized complete block design. Tukey’s multiple comparison procedures were used to detect and separate the eighteen means differences at *p* < 0.05. Pearson’s correlation coefficient was used for the determination of the relationships between the variables by using SPSS Statistics 21.0 software (SPSS Inc., Chicago, IL, USA). The web tool Clustvis [73] was used for the visualization of clustering on multivariate data using PCA and AHC. The construction of two-dimensional (2-D) plots was based on the first two principal components (PCs). The AHC analysis was performed using Euclidean distance and Ward’s method for agglomeration to systematically analyze the combined bioactive compounds and antioxidant capacity per *C. maritimum* genotype.

Principal Coordinate Analysis (PCoA), which is used when variables are qualitative or discrete, thus offering a unique analytical solution, was applied for molecular data. PCoA based on Euclidean distance was performed using the cmdscale function in R 4.1.0 [74]. For the identification of unique genotypes of *C. maritimum*, all possible pairwise comparisons between two genotypes were attempted. The number of alleles per locus (Na) and the observed (Ho) and expected (He) heterozygosity (assuming Hardy–Weinberg equilibrium) were calculated using GenAlEx software [75]. The probability that two individuals will, by chance, have the same multilocus genotype was investigated by estimates for Probability of Identity (PI) and Probability of Identity between siblings (PIsibs), which were also inferred with the same software. Polymorphic Information Content (PIC) for each genetic marker employed was estimated by the polysat R package [76], whereas UPGMA dendrograms were constructed. Three different genetic distances, namely relative dissimilarity, Euclidean distance, and the individual genetic distance measure of Smouse and Peakall were used [77].

Possible population structure was analyzed to examine how geographical distribution affects the genotype similarity in genetically homogenous populations using a model-based Bayesian procedure implemented in the STRUCTURE v.2.3.4 software [78,79]. Genotypes were divided into genetic clusters using the “admixture” model along with the “uncorrelated allele frequencies” model. Analyses were run with 500,000 burn-in iterations followed by 1,000,000 iterations for Markov chain Monte Carlo in 20 independent runs for each number of clusters (K) from 1 to 5. The most likely K was determined by employing the Evanno method [49] and visualizations were made using the pophelper v2.3.1 R package [80].

## 4. Conclusions

In summary, the present work constitutes the first characterization of native Greek rock samphire germplasm in terms of metabolomic and molecular fingerprinting. The wide variation observed in the bioactive compounds and the antioxidant capacity, as well as the low variability and the low to moderate diversity observed in the genetic profile of Greek rock samphire genotypes, revealed that the germplasm collection conserved in the Balkan Botanic Garden of Kroussia in Northern Greece could serve as an important source of genetic material for artificial selection and future plant breeding. Interestingly, the most remarkable and distinctive genotype genotypes were G9 from Kefalonia Island, followed by G4 from Ikaria Island; both clearly exhibited a rather similar response with high values in the evaluated traits. Based on the reported outcomes here, these *C. maritimum* genotypes stand out as well-documented, and therefore they can be prioritized for large scale cultivation. Additionally, these genotypes can be the springboard for the development of new *C. maritimum* cultivars with desirable traits with respect to polyphenolic content and/or antioxidant potential.

## Figures and Tables

**Table 1:** Origin of the 18 native Greek rock samphire genotypes studied, with their IPEN (International Plant Exchange Network) accession numbers and their abbreviations.

a/a	IPEN Accession Number	Genotype Abbreviation	Origin in Greece
1	GR-1-BBGK-17,6006	G1	Karavostamo, Ikaria Island
2	GR-1-BBGK-16,5963	G2	Kalamaki, Tymbaki, Heraklion, Crete
3	GR-1-BBGK-12,5685_A	G3	Karavostamo, Ikaria Island
4	GR-1-BBGK-12,5685_B	G4	Karavostamo, Ikaria Island
5	GR-1-BBGK-12,5685_C	G5	Karavostamo, Ikaria Island
6	GR-1-BBGK-16,5962	G6	Heraklion city, Crete
7	GR-1-BBGK-16,5961	G7	Vatopedi, Agio Oros, Chalkidiki
8	GR-1-BBGK-16,5964	G8	Rethimno city, Crete
9	GR-1-BBGK-17,5972	G9	Kefalonia, Ionian Islands
10	GR-1-BBGK-16,5965	G10	Kalamata, Peloponnese
11	GR-1-BBGK-05,3041	G11	Kefalonia, Ionian Islands
12	GR-1-BBGK-97,719_A	G12	Agio Oros, Chalkidiki
13	GR-1-BBGK-97,719	G13	Agio Oros, Chalkidiki
14	GR-1-BBGK-14,5800	G14	Pantana, Heraklion, Crete
15	GR-1-BBGK-15_5902_A	G15	Ikaria Island
16	GR-1-BBGK-15,5902	G16	Ikaria Island
17	GR-1-BBGK-05,3001	G17	Chania city, Crete
18	GR-1-BBGK-10,5458	G18	Vokaria, Chios Island

**Table 2:** Results of an analysis of variance applied on the evaluated bioactive compounds, antioxidant capacity parameters, and phenolic groups identified by an LC-MS analysis of the aboveground portion of 18 native Greek rock samphire genotypes. F-ratios’ significance is given for the effects exerted by replication and rock samphire genotype.

Significance of *F*-Ratio											
								Determined by LC-MS			
Variation Source	df ^z^	ABTS	DPPH	FRAP	AsA	TPC	TFC	CQAs	OPAs	FLAs	Total
**Replication**	2	NS	NS	NS	NS	NS	NS	NS	NS	NS	NS
**Genotype**	17	***	***	***	***	***	***	***	***	***	***
**CV%**		8.68	9.49	10.35	17.5	5.63	7.80	4.58	5.82	5.96	4.14

^z^ df, degrees of freedom; ABTS, 2,2-azino-bis-(3-ethylbenzothiazoline-6-sulfonic acid); DPPH, 2,2-diphenyl-1-picrylhydrazyl radical scavenging activity; FRAP, ferric reducing antioxidant power; AsA, ascorbic acid content; TPC, total phenolic content; TFC, total flavonoid content; CQAs, caffeolquinic acids; OPAs, other phenolic acids; FLAs, flavonoids; Total, total phenolics; CV, coefficient of variance; ***, significance at *p* < 0.001; NS, non-significant.

**Table 3:** Eighteen main phenolic compounds with their abbreviations, identified by HPLC-DAD-MS in methanolic extracts of native Greek rock samphire genotypes.

Peak	R_t_ (Min)	λ_max_ (nm)	[M-H]^−^ (m/z)	Attempt to Identify	Abbreviation
1	2.78	330	191	Quinic acid	QNA
2	4.01	325	353	1-caffeoyl-quinic acid	1-CQA
3	4.34	325	353	5-O-caffeoylquinic acid	5-CQA
4	5.01	260	153	Protocatechuic acid	PRCA
5	5.65	325	353	3-O-caffeoylquinic acid	3-CQA
6	5.85	325	353	4-O-caffeoylquinic acid	4-CQA
7	6.36	270, 335	593	Vicenin-2	VIC
8	7.51	311	337	5-coumaroyl-quinic acid	5-cQA
9	7.61	320	179	Caffeic acid	CA
10	8.24	297, 325	367	5-feruloy-quinic acid	5-fQA
11	8.84	311	337	5-coumaroyl-quinic acid isomer	5-cQA
12	9.21	256, 354	609	Quercetin-3-O-rutinoside	QURU
13	9.63	260, 36	463	Quercetin-3-O-glucoside	QUGL
14	9.78	260, 36	463	Quercetin-3-O-galactoside	QUGA
15	9.98	309	163	*p*-coumaric acid	pCA
16	10.45	327	515	3,4-dicaffeoyl-quinic acid	3,4-DCQA
17	10.95	327	515	3,5-dicaffeoyl-quinic acid	3,5-DCQA
18	11.61	327	515	4,5-dicaffeoul quinic acid	4,5-DCQA

**Table 4:** Content (mg/100 g of dw) of main caffeolquinic acids (CQAs) of 18 native Greek rock samphire genotypes (for abbreviations and origin, see Table 1). Different letters in the same column characterize significant differences among genotypes according to Tukey’s test for *p* ≤ 0.05.

Genotype	CQAs																			
	1-CQA		5-CQA		3-CQA		4-CQA		5-cQA		5-fQA		3,4-DCQA		3,5-DCQA		4,5-DCQA		Total	
G1	112.70	defg	16.02	ef	310.05	bc	45.95	a	474.79	abc	87.41	abcd	15.39	fghi	259.54	bc	12.83	d	1334.68	b
G2	125.33	cde	44.01	a	298.60	bc	31.43	b	488.84	ab	71.21	defg	15.10	fghi	259.25	bc	10.57	de	1344.34	b
G3	147.06	abc	14.99	ef	315.45	bc	22.97	e	361.85	efg	85.82	abcd	17.75	cdef	282.04	ab	13.03	d	1260.96	b
G4	130.28	bcd	37.23	b	286.16	bcd	32.45	b	358.99	efg	91.66	abcd	24.00	a	227.95	cde	19.71	ab	1208.43	bcd
G5	148.05	abc	26.88	c	296.71	bc	26.07	d	399.60	def	83.76	abcde	19.38	bcde	218.75	de	17.36	bc	1236.56	bc
G6	112.43	defg	37.01	b	251.24	def	23.42	e	346.02	fgh	81.30	bcdef	16.82	efg	208.85	ef	7.89	f	1084.98	cde
G7	137.09	bcd	13.05	fgh	299.81	bc	28.87	c	446.51	bcd	72.80	cdefg	16.11	fgh	226.85	de	12.02	d	1253.11	b
G8	61.90	h	20.30	de	305.51	bc	18.41	f	488.46	ab	88.77	abc	20.19	bcd	241.27	cd	16.30	c	1261.11	b
G9	171.86	abc	25.18	cd	372.65	a	24.61	de	520.49	a	100.96	a	22.46	ab	291.40	a	17.68	abc	1547.29	a
G10	157.01	abc	24.39	cd	288.83	bcd	26.82	cd	416.57	cde	83.43	bcdef	20.79	abc	223.18	dc	17.60	bc	1258.62	b
G11	121.41	cdef	7.79	hi	277.11	cde	17.30	fg	329.62	gh	63.90	fg	12.42	i	231.55	cde	8.65	ef	1069.75	de
G12	110.66	defg	14.63	efg	227.57	f	15.32	g	280.56	hi	79.34	bcdefg	12.28	i	165.20	g	7.26	f	912.82	f
G13	25.83	i	3.61	i	83.97	g	5.58	j	208.54	j	26.58	h	3.08	k	55.15	h	1.75	g	414.09	g
G14	92.32	g	11.32	fgh	236.84	f	18.07	f	278.14	hi	70.94	defg	17.24	def	239.49	cde	20.14	a	984.50	ef
G15	91.24	g	7.58	hi	230.40	f	9.83	i	287.10	hi	67.70	efg	8.86	j	179.57	fg	6.75	f	889.03	f
G16	28.13	i	3.59	i	79.20	g	3.20	j	233.39	ij	28.92	h	3.51	k	60.91	h	3.13	g	443.98	g
G17	100.80	efg	11.99	fgh	251.39	def	22.33	e	367.18	efg	93.84	ab	13.47	hi	213.77	de	7.69	f	1082.46	cde
G18	93.83	fg	9.03	ghi	245.66	ef	12.22	h	358.03	efg	63.66	g	13.84	ghi	214.09	de	12.53	d	1022.89	ef
Mean value	109.32		18.26		258.73		21.38		369.15		74.56		15.15		211.05		11.83		1089.42	

**Table 5:** Content (mg/100 g of dw) of flavonoids (FLAs) of 18 native Greek rock samphire genotypes (for abbreviations and origin, see Table 1). Different letters in the same column characterize significant differences among genotypes according to Tukey’s test for *p* ≤ 0.05.

Genotype	FLAs									
	VIC		QURU		QUGL		QUGA		Total	
G1	13.97	f	29.21	bc	0.82	fgh	2.71	def	46.71	bcd
G2	20.59	bc	33.68	ab	7.88	b	4.81	abc	66.96	a
G3	14.40	f	22.57	def	1.46	fg	3.55	bcde	41.98	de
G4	25.32	a	35.08	ab	2.97	cd	5.88	a	69.25	a
G5	19.28	bcd	28.58	bc	1.05	fgh	4.46	abc	53.37	b
G6	21.94	b	23.85	cde	2.88	cd	3.29	cde	51.96	b
G7	13.41	f	18.97	efg	2.99	cd	2.38	efg	37.75	ef
G8	21.80	bv	17.44	fg	1.79	ef	2.62	efg	43.65	cde
G9	16.06	ef	25.70	cde	18.27	a	5.11	abc	65.14	a
G10	18.92	cde	24.95	cde	3.87	c	3.36	cde	51.1	bc
G11	8.21	g	10.38	ij	2.54	de	1.26	fg	22.39	hi
G12	2.53	h	10.14	ij	1.06	fgh	1.15	fg	14.88	ij
G13	4.14	h	5.77	j	0.47	gh	1.01	g	11.39	j
G14	8.28	g	14.58	ghi	2.50	de	2.73	def	28.09	gh
G15	10.27	g	10.51	hij	0.62	gh	1.41	fg	22.81	h
G16	17.49	de	16.10	ghi	0.18	h	3.73	bcde	37.5	ef
G17	8.84	g	20.11	defg	0.88	fgh	4.00	bcde	33.83	fg
G18	9.62	g	18.10	fg	0.75	gh	4.34	abcd	32.81	fg
Mean value	14.17		20.32		2.94		3.21		40.64	

**Table 6:** Content (mg/100 g of dw) of other phenolic acids (OPAs) of 18 native Greek rock samphire genotypes (for abbreviations and origin, see Table 1). Different letters in the same column characterize significant differences among genotypes according to Tukey’s test for *p* ≤ 0.05.

Genotype	OPAs									
	QNA		PRCA		CA		pCA		Total	
G1	105.75	a	0.42	de	0.61	fg	0.06	g	106.84	a
G2	74.48	efghi	0.41	def	0.72	defg	0.12	efg	75.73	defgh
G3	73.33	fghi	0.60	bc	0.63	efg	0.07	fg	74.63	defgh
G4	86.76	cdefg	0.41	def	0.86	cdef	0.08	fg	88.11	bcde
G5	80.64	defgh	0.47	cd	0.88	cdef	0.20	cde	82.19	cdef
G6	71.51	ghij	0.51	bcd	0.99	bc	0.32	b	73.33	efgh
G7	101.27	abcd	0.57	bc	0.69	defg	0.09	fg	102.62	ab
G8	60.73	ij	0.63	b	0.79	cdefg	0.30	b	62.45	gh
G9	89.57	bcde	0.50	bcd	1.49	a	0.24	bcd	91.8	abc
G10	104.90	ab	0.77	a	0.88	cde	0.16	def	106.71	a
G11	95.72	abcd	0.60	bc	1.15	bc	0.13	efg	97.6	ab
G12	92.03	abcd	0.31	ef	1.01	bc	0.07	fg	93.42	abc
G13	74.32	efghij	0.85	a	0.65	defg	0.56	a	76.38	defg
G14	70.92	hij	0.50	bcd	0.63	efg	0.08	fg	72.13	fgh
G15	88.16	cdefg	0.28	f	0.90	bcd	0.29	bcd	89.63	bcd
G16	90.47	abcd	0.46	cd	0.59	g	0.53	a	92.05	abc
G17	59.02	j	0.50	bcd	1.01	bc	0.31	b	60.84	h
G18	71.44	ghij	0.39	def	0.72	defg	0.10	efg	72.65	fgh
Mean value	82.83		0.51		0.84		0.21		84.40	

**Table 7:** Correlation matrix (r coefficients) between bioactive compounds, antioxidant capacity, and phenolic groups identified by LC-MS and AsA content in 18 native Greek rock samphire genotypes.

Parameters	TPC ^z^	TFC	ABTS	DPPH	FRAP	CQAs	OPAs	FLAs	AsA
TPC	1	0.992 ***	0.868 ***	0.955 ***	0.977 ***	0.897 ***	0.102	0.649 ***	0.332
TFC		1	0.880 ***	0.949 ***	0.964 ***	0.892 ***	0.141	0.633 ***	0.317
ABTS			1	0.882 ***	0.828 ***	0.815 ***	0.051	0.559 *	0.420
DPPH				1	0.949 ***	0.907 ***	0.066	0.727 ***	0.241
FRAP					1	0.879 ***	0.057	0.670 **	0.277
CQAs						1	0.113	0.691 **	0.150
OPAs							1	0.047	0.027
FLAs								1	−0.019
AsA									1

^**z**^ TPC, total phenolic content; TFC, total flavonoid content; ABTS, 2,2-azino-bis-(3-ethylbenzothiazoline-6-sulfonic acid); DPPH, 2,2-diphenyl-1-picrylhydrazyl radical scavenging activity; FRAP, ferric reducing antioxidant power; CQAs, caffeolquinic acids; OPAs, other phenolic acids; FLAs, flavonoids; AsA, Ascorbic acid. Asteriks, *** indicate significant at *p* ≤ 0.001, ** at *p* ≤ 0.01, and * at *p* ≤ 0.05.

**Table 8:** Diversity statistics for the 18 native Greek rock samphire genotypes based on GenAlEx analysis for allelic patterns.

Locus	Na	Ne	I	Ho	uHe	PI	PIsibs	PIC
CM14	3.000	2.249	0.907	0.529	0.572	0.282	0.543	0.469
CM15	7.000	5.635	1.833	0.722	0.846	0.055	0.352	0.8
CM33	6.000	3.846	1.559	1.000	0.766	0.098	0.405	0.714
CM34	2.000	1.117	0.215	0.111	0.108	0.807	0.899	0.099
CM03	3.000	1.476	0.572	0.278	0.332	0.496	0.713	0.285
CM04	6.000	1.846	0.999	0.389	0.471	0.315	0.599	0.437
CM11	7.000	2.769	1.359	0.667	0.657	0.164	0.472	0.605
CM12	10.000	6.113	2.004	0.611	0.860	0.046	0.343	0.817
Mean	5.500	3.131	1.181	0.538	0.577	0.283	0.541	0.528

## Data Availability

The data is available from the authors upon request.

## References

[1] Sarrou E., Siomos A.S., Riccadona S., Aktsoglou D.-C., Tsouvaltzis P., Angeli A., Franceschi P., Chatzopoulou P., Vrhovsek U., Martens S. (2019). Improvement of sea fennel (*Crithmum maritimum* L.) nutritional value through iodine biofortification in a hydroponic floating system. Food Chem..

[2] Pateira L., Nogueira T., Antunes A., Venâncio F., Tavares R., Capelo J. (1999). Two chemotypes of *Crithmum maritimum* L. from Portugal. Flavour Fragr. J..

[3] Atia A., Barhoumi Z., Mokded R., Abdelly C., Smaoui A. (2011). Environmental eco-physiology and economical potential of the halophyte *Crithmum maritimum* L. (Apiaceae). J. Med. Plants Res..

[4] Hamed K.B., Debez A., Chibani F., Abdelly C. (2004). Salt response of *Crithmum maritimum*, an oleaginous halophyte. Trop. Ecol..

[5] Latron M., Arnaud J.F., Ferla H., Godé C., Duputié A. (2018). Polymorphic nuclear markers for coastal plant species with dynamic geographic distributions, the rock samphire (*Crithmum maritimum*) and the vulnerable dune pansy (*Viola tricolor* subsp. curtisii). Mol. Biol. Rep..

[6] Renna M. (2018). Reviewing the prospects of sea fennel (*Crithmum maritimum* L.) as emerging vegetable crop. Plants.

[7] Özcan M., Akgül A., Başcr K.H.C., Özck T., Tabanca N. (2001). Essential oil composition of sea fennel (*Crithmum maritimum*) form Turkey. Nahrung Food.

[8] Pereira A.G., Fraga-Corral M., García-Oliveira P., Jimenez-Lopez C., Lourenço-Lopes C., Carpena M., Otero P., Gullón P., Prieto M.A., Simal-Gandara J. (2020). Culinary and nutritional value of edible wild plants from northern Spain rich in phenolic compounds with potential health benefits. Food Funct..

[9] Giungato P., Renna M., Rana R., Licen S., Barbieri P. (2019). Characterization of dried and freeze-dried sea fennel (*Crithmum maritimum* L.) samples with headspace gas-chromatography/mass spectrometry and evaluation of an electronic nose discrimination potential. Food Res. Int..

[10] Montesano F.F., Gattullo C.E., Parente A., Terzano R., Renna M. (2018). Cultivation of potted sea fennel, an emerging Mediterranean halophyte, using a renewable seaweed-based material as a peat substitute. Agriculture.

[11] Zarrouk M., El Almi H., Ben Youssef N., Sleimi N., BenMiled D., Smaoui A., Abdelly C., Lieth H. (2003). Lipid composition of seeds of local halophytes: *Cakile maritima*, *Zygophyllum album* and *Crithmum maritimum*. Cash Crop Halophytes: Recent Studies.

[12] Jallali I., Megdiche W., M’Hamdi B., Queslati S., Smaoui A., Abdelly C., Ksouri R. (2012). Changes in phenolic composition and antioxidant activities of the edible halophyte *Crithmum maritimum* L. with physiological stage and extraction method. Acta Physiol Plant.

[13] Jallali I., Zaouali Y., Missaoui I., Smeoui A., Abdelly C., Ksouri R. (2014). Variability of antioxidant and antibacterial effects of essential oils and acetonic extracts of two edible halophytes: *Crithmum maritimum* L. and *Inula crithmoїdes* L.. Food Chem..

[14] Meot-Duros L., Magné C. (2009). Antioxidant activity and phenol content of *Crithmum maritimum* L. leaves. Plant Physiol. Biochem..

[15] Suresh U., Murugan K., Panneerselvam C., Aziz Al T., Cianfaglione K., Wang L., Maggi F. (2020). Encapsulation of sea fennel (*Crithmum maritimum*) essential oil in nanoemulsion and SiO_2_ nanoparticles for treatment of the crop pest *Spodoptera litura* and the dengue vector *Aedes aegypti*. Ind. Crops Prod..

[16] Mekinić G.I., Blažević I., Mudnić I., Burčul F., Grga M., Skroza D., Jerčić I., Ljubenkov I., Boban M., Miloš M. (2016). Sea fennel (*Crithmum maritimum* L.): Phytochemical profile, antioxidative, cholinesterase inhibitory and vasodilatory activity. J. Food Sci. Technol..

[17] Burczyk J., Wierzchowska-Renke K., Głowniak K., Głowniak P., Marek D. (2002). Geographic and environmental influences on the variation of essential oil and coumarins in *Crithmum maritimum* L.. J. Herbs Spices Med. Plants.

[18] Sánchez-Faure A., Calvo M.M., Pérez-Jiménez J., Martín-Diana A.B., Rico D., Montero M.P., Gómez-Guillén M.C., López-Caballero M.E., Martínez-Alvarez O. (2020). Exploring the potential of common iceplant, seaside arrowgrass and sea fennel as edible halophytic plants. Food Res. Int..

[19] Grigoriadou K., Maloupa E. (2008). Micropropagation and salt tolerance of in vitro grown *Crithmum maritimum* L.. Plant Cell Tissue Organ Cult..

[20] Meot-Duros L., Magné C. (2008). Effect of salinity and chemical factors on seed germination in the halophyte *Crithmum maritimum* L.. Plant Soil.

[21] Kadereit J., Arafeh R., Somogyi G., Westberg E. (2005). Terrestrial growth and marine dispersal? Comparative phylogeography of five coastal plant species at a European scale. Taxon.

[22] Arafeh R.M. (2005). Molecular Phylogeography of the European Coastal Plants *Crithmum maritimum* L., *Halimione portulacoides* (L.) Aellen, *Salsola kali* L. and *Calystegia soldanella* (L.) R.Br. Ph.D. Thesis.

[23] Xanthopoulou A., Ganopoulos I., Kalivas A., Nianiou-Obeidat I., Ralli P., Moysiadis T., Tsaftaris A., Madesis P. (2015). Comparative analysis of genetic diversity in Greek Genebank collection of summer squash (*‘Cucurbita pepo’*) landraces using start codon targeted (SCoT) polymorphism and ISSR markers [online]. Aust. J. Crop Sci..

[24] Vos P., Hogers R., Bleeker M., Reijans M., van de Lee T., Hornes M., Friters A., Pot J., Paleman J., Kuiper M. (1995). AFLP: A new technique for DNA fingerprinting. Nucleic Acids Res..

[25] Roncallo P.F., Beaufort V., Larsen A.O., Dreisigacker S., Echenique V. (2019). Genetic diversity and linkage disequilibrium using SNP (KASP) and AFLP markers in a worldwide durum wheat (*Triticum turgidum* L. var. *durum*) collection. PLoS ONE.

[26] Stathi Ε., Kougioumoutzis Κ., Abraham Ε.Μ., Trigas P., Ganopoulos I., Avramidou E.V., Tani E. (2020). Population genetic variability and distribution of the endangered Greek endemic *Cicer graecum* under climate change scenarios. AoB Plants.

[27] Zietkiewicz E., Rafalski J., Labuda D. (1994). Genome fingerprinting by simple sequence repeat (SSR)-anchored polymerase chain reaction amplification. Genomics.

[28] Ganopoulos I., Merkouropoulos G., Pantazis S., Tsipouridis C., Tsaftaris A. (2011). Assessing molecular and morpho-agronomical diversity and identification of ISSR markers associated with fruit traits in quince (*Cydonia oblonga*). Gen. Mol. Res..

[29] Nabet N., Boudries H., Chougui N., Loupassaki S., Souagui S., Burló F., Hernández F., Carbonell-Barrachina Á.A., Madani K., Larbat R. (2017). Biological activities and secondary compound composition from *Crithmum maritimum* aerial parts. Int. J. Food Prop..

[30] Sousa G., Alves M.I., Neves M., Tecelão C., Ferreira-Dias S. (2022). Enrichment of sunflower oil with ultrasound-assisted extracted bioactive compounds from *Crithmum maritimum* L.. Foods.

[31] Souid A., Della Croce C.M., Frassinetti S., Gabriele M., Pozzo L., Ciardi M., Abdelly C., Hamed K.B., Magné C., Longo V. (2021). Nutraceutical potential of leaf hydro-ethanolic extract of the edible halophyte *Crithmum maritimum* L.. Molecules.

[32] Males Z., Zuntar I., Nigovic B., Plazibat M., Vundać V.B. (2003). Quantitative analysis of the polyphenols of the aerial parts of rock samphire-*Crithmum maritimum* L.. Acta Pharm..

[33] Petropoulos S.A., Karkanis A., Martins N., Ferreira I.C. (2018). Edible halophytes of the Mediterranean basin: Potential candidates for novel food products. Trends Food Sci. Technol..

[34] Chu Y.F., Sun J., Wu X., Liu R.H. (2002). Antioxidant and antiproliferative activities of common vegetables. J. Agric. Food Chem..

[35] Martins-Noguerol R., Matías L., Pérez-Ramos I.M., Moreira X., Muñoz-Vallés S., Mancilla-Leytón J.M., Francisco M., García-González A., DeAndrés-Gil C., Martínez-Force E. (2022). Differences in nutrient composition of sea fennel (*Crithmum maritimum*) grown in different habitats and optimally controlled growing conditions. J. Food Compos. Anal..

[36] Zhou K., Yu L. (2006). Total phenolic contents and antioxidant properties of commonly consumed vegetables grown in Colorado. LWT-Food Sci. Technol..

[37] Pereira C.G., Barreira L., da Rosa Neng N., Nogueira J.M.F., Marques C., Santos T.F., Varela J., Custódio L. (2017). Searching for new sources of innovative products for the food industry within halophyte aromatic plants: In vitro antioxidant activity and phenolic and mineral contents of infusions and decoctions of *Crithmum maritimum* L.. Food Chem. Toxicol..

[38] Zafeiropoulou V., Tomou E.M., Ioannidou O., Karioti A., Skaltsa H. (2020). Sea fennel: Phytochemical analysis of Greek wild and cultivated *Crithmum maritimum* L. populations, based on HPLC-PDA-MS and NMR methods. J. Pharmacogn. Phytochem..

[39] Alves-Silva J.M., Guerra I., Gonçalves M.J., Cavaleiro C., Cruz M.T., Figueirinha A., Salgueiro L. (2020). Chemical composition of *Crithmum maritimum* L. essential oil and hydrodistillation residual water by GC-MS and HPLC-DAD-MS/MS, and their biological activities. Ind. Crops Prod..

[40] Siracusa L., Kulisic-Bilusic T., Politeo O., Krause I., Dejanovic B., Ruberto G. (2011). Phenolic cmposition and antioxidant activity of aqueous infusions from *Capparis spinosa* L. and *Crithmum maritimum* L. before and after submission to a two-step in vitro digestion model. J. Agric. Food Chem..

[41] Mekinić I.G., Šimat V., Ljubenkov I., Burčul F., Grga M., Mihajlovski M., Lončar R., Katalinić V., Skroza D. (2018). Influence of the vegetation period on sea fennel, *Crithmum maritimum* L. (Apiaceae), phenolic composition, antioxidant and anticholinesterase activities. Ind. Crops Prod..

[42] Schaller R.G., Schnitzler W.H. (2000). Nitrogen nutrition and flavour compounds of carrots (*Daucus carota* L.) cultivated in Mitscherlich pots. J. Sci. Food Agric..

[43] Brooks J.S., Feeny P. (2004). Seasonal variation in *Daucus carota* leaf-surface and leaf tissue chemical profile. Biochem. Syst. Ecol..

[44] Krizman M., Baricevic D., Prosek M. (2007). Determination of phenolic compounds in fennel by HPLC and HPLC-MS using a monolithic reversed-phase column. J. Pharm. Biomed. Anal..

[45] Vina S.Z., Chaves A.R. (2006). Antioxidant responses in minimally processed celery during refrigerated storage. Food Chem..

[46] Irakli M., Skendi A., Bouloumpasi E., Chatzopoulou P., Biliaderis C.G. (2021). LC-MS identification and quantification of phenolic compounds in solid residues from the essential oil industry. Antioxidants.

[47] Xu J.G., Hu Q.P., Liu Y. (2012). Antioxidant and DNA-protective activities of chlorogenic acid isomers. J. Agric. Food Chem..

[48] Kooti W., Moradi M., Ali-Akbari S., Sharafi-Ahvazi N., Asadi-Samani M., Ashtary-Larky D. (2015). Therapeutic and pharmacological potential of *Foeniculum vulgare* Mill: A review. J. Herbmed Pharmacol..

[49] Evanno G., Regnaut S., Goudet J. (2005). Detecting the number of clusters of individuals using the software Structure: A simulation study. Mol. Ecol..

[50] Weising K., Freitag H. (2007). Phylogeography of halophytes from European coastal and inland habitats. Zool. Anz..

[51] Templeton A.R. (2007). Genetics and recent human evolution. Evolution.

[52] Garcia A.A.F., Benchimol L.L., Barbosa A.M.M., Geraldi I.O., Souza C.L., de Souza A.P. (2004). Comparison of RAPD, RFLP, AFLP and SSR markers for diversity studies in tropical maize inbred lines. Genet. Mol. Biol..

[53] Saha M.C., Mian M.A.R., Eujayl I., Zwonitzer J.C., Wang L., May G.D. (2004). Tall fescue EST-SSR markers with transferability across several grass species. TAG.

[54] Rani J., RaShmi K., Sinha S., Sahay S., Mandal S.S., Singh B. (2020). Molecular diversity of babycorn (*Zea mays*) inbred lines by rice SSR Marker. Indian J. Agric. Sci..

[55] Sara S.M., Said W.M., Abdel-Tawab F.M., Kamal L.M., Mahmoud Shata S. (2021). Phylogenetic relationships of some barley (*Hordeum vulgare* L.) accessions in Egypt. EJGC.

[56] Song Y., Chen Y., Lv J., Xu J., Zhu S., Li M. (2019). Comparative chloroplast genomes of *Sorghum* Species: Sequence divergence and phylogenetic relationships. Biomed Res. Int..

[57] Tripathi S., Singh S.K., Srivashtav V., Khaire A.R., Vennela P., Singh D.K. (2020). Molecular diversity analysis in rice (*Oryza sativa* L.) using SSR markers. Electron. J. Plant Breed..

[58] Escudero M., Vargas P., Arens P., Ouborg N.J., LuceÑo M. (2010). The east-west-north colonization history of the Mediterranean and Europe by the coastal plant *Carex extensa* (Cyperaceae). Mol. Ecol..

[59] Lu L., Li X., Hao Z., Yang L., Zhang J., Peng Y., Xu H., Lu Y., Zhang J., Shi J. (2017). Phylogenetic studies and comparative chloroplast genome analyses elucidate the basal position of halophyte *Nitraria sibirica* (Nitrariaceae) in the Sapindales. DNA Mapp. Seq. Anal..

[60] Mondal T.K., Rawal H.C., Chowrasia S., Varshney D., Panda A.K., Mazumdar A., Kaur H., Gaikwad K., Sharma T.R., Singh N.K. (2018). Draft genome sequence of first monocot-halophytic species *Oryza coarctata* reveals stress-specific genes. Sci. Rep..

[61] Nayak S.S., Pradhan S., Sahoo D., Parida A. (2020). De novo transcriptome assembly and analysis of *Phragmites karka*, an invasive halophyte, to study the mechanism of salinity stress tolerance. Sci. Reps..

[62] Kadereit J.W., Westberg E. (2007). Phylogeographic structure of seven coastal species. Determinants of phylogeographic structure: A comparative study of seven coastal flowering plant species across their European range. Watsonia.

[63] James F.C., Mcculloch C.E. (1990). Multivariate analysis in ecology and systematics: Panacea or pandora’s box?. Annu. Rev. Ecol. Sys..

[64] Schlee D., Sneath P.H.A., Sokal R.R., Freeman W.H. (1975). Numerical taxonomy. The principles and practice of numerical classification. Syst. Zool..

[65] Frankel E.N., Meyer A.S. (2000). The problems of using one-dimensional methods to evaluate multifunctional food and biological antioxidants. J. Sci. Food Agric..

[66] Re R., Pellegrini N., Proteggente A., Pannala A., Yang M., Rice-Evans C. (1999). Antioxidant activity applying an improved ABTS radical cation decolorization assay. Free Radic. Biol. Med..

[67] Yen G.C., Chen H.Y. (1995). Antioxidant activity of various tea extracts in relation to their antimutagenicity. J. Agric. Food Chem..

[68] Benzie F., Strain J. (1999). Ferric reducing/antioxidant power assay: Direct measure of total antioxidant activity of biological fluids and modified version for simultaneous measurement of total antioxidant power and ascorbic acid concentration. Methods Enzymol..

[69] Pateraki I., Sanmartin M., Kalamaki M.S., Gerasopoulos D., Kanellis A.K. (2004). Molecular characterization and expression studies during melon fruit development and ripening of L-galactono-1,4-lactone dehydrogenase. J. Exp. Bot..

[70] Singleton V.L., Orthofer R., Lamuela-Raventós R.M. (1999). Analysis of total phenols and other oxidation substrates and antioxidants by means of Folin-Ciocalteu reagent. Meth. Enzymol..

[71] Irakli M.N., Samanidou V.F., Biliaderis C.G., Papadoyannis I.N. (2012). Development and validation of an HPLC-method for determination of free and bound phenolic acids in cereals after solid-phase extraction. Food Chem..

[72] Bao S., Cai Y., Sun M., Wang G.Y., Corke H. (2005). Anthocyanins, flavonols, and free radical scavenging activity of Chinese bayberry (*Myrica rubra*) extracts and their color properties and stability. J. Agric. Food Chem..

[73] Metsalu T., Vilo J. (2015). ClustVis: A web tool for visualizing clustering of multivariate data using Principal Component Analysis and heatmap. Nucleic Acids Res..

[74] R Core Team (2021). R: A Language and Environment for Statistical Computing.

[75] Peakall R., Smouse P.E. (2012). GenAlEx 6.5: Genetic analysis in Excel. Population genetic software for teaching and research—An update. Bioinformatics.

[76] Clark L.V., Jasieniuk M. (2011). Polysat: An R package for polyploid microsatellite analysis. Mol. Ecol. Resour..

[77] Smouse P.E., Peakall R. (1999). Spatial autocorrelation analysis of individual multiallele and multilocus genetic structure. Heredity.

[78] Baldoni L., Tosti N., Ricciolini C., Belaj A., Arcioni S., Pannelli G., Germana M.A., Mulas M., Porceddu A. (2006). Genetic structure of wild and cultivated olives in the Central Mediterranean Basin. Ann. Bot..

[79] Rosenberg N.A., Pritchard J.K., Weber J.L., Cann H.M., Kidd K.K., Zhivotovsky L.A., Feldman M.W. (2002). Genetic structure of human populations. Science.

[80] Francis R.M. (2017). Pophelper: An R package and web app to analyse and visualize population structure. Mol. Ecol. Resour..

